# Remdesivir in COVID‐19: A Focus on Pediatric Cardiac Patients

**DOI:** 10.1155/cjid/4700812

**Published:** 2026-02-24

**Authors:** Dima Bsat, Dalia Safi, Mariam Arabi

**Affiliations:** ^1^ Faculty of Medicine, American University of Beirut Medical Center, Beirut, Lebanon, aubmc.org.lb; ^2^ Department of Pediatric and Adolescent Medicine, Division of Pediatric Cardiology, American University of Beirut Medical Center, Beirut, Lebanon, aubmc.org.lb

**Keywords:** coronavirus, COVID-19, remdesivir, SARS-CoV-2

## Abstract

The coronavirus disease 2019 (COVID‐19) pandemic has presented a significant global health challenge that necessitated the immediate search for various therapeutic modalities. Remdesivir, an antiviral drug inhibiting RNA‐dependent RNA polymerase (RdRp), was among the most heavily used drugs against COVID‐19. Of the several randomized controlled trials studying the efficacy of remdesivir, the vast majority were studied on the adult population. Results remain contradictory, with some studies supporting the high efficacy of remdesivir while others highlighting the lack of significance of its antiviral effects. Given the lack of focus on the pediatric population, the antiviral effects of remdesivir in cardiac pediatric patients, who are particularly vulnerable, remain especially under‐investigated. This literature review explores current literature on remdesivir’s mechanism of action and efficacy against COVID‐19, especially in the pediatric cardiac population. Therefore, by combining results of studies from randomized controlled trials and retrospective studies in the adult and pediatric populations, this literature review underlines current knowledge gaps and highlights the need for studies targeting specific ages and comorbidities to effectively treat patients at higher risk of adverse events.

## 1. Introduction

First observed in Wuhan, China, the severe acute respiratory syndrome coronavirus 2 (SARS‐CoV‐2) led to the coronavirus disease 2019 (COVID‐19) pandemic. SARS‐CoV‐2 is a single‐stranded RNA virus under the Coronaviridae family and the Nidovirales order [1]. Given its rapid spread, more than 770 million COVID‐19 cases and 7 million deaths were reported due to COVID‐19 as of April 2025, leading to global fright [2]. Unlike the common cold coronaviruses that cause mild symptoms, SARS‐CoV‐2 causes severe flu‐like symptoms that can lead to severe systemic effects, including acute respiratory distress syndrome (ARDS), pneumonia, and acute kidney injury (AKI). The symptoms of COVID‐19 have progressed past respiratory and gastrointestinal effects, causing long‐term consequences like myocardial inflammation [3], neuroinflammation [4], and lung fibrotic abnormalities [5].

While the impact of the COVID‐19 pandemic has decreased, the virus is still circulating globally. Patients with congenital heart disease (CHD) have an increased risk of severe outcomes with COVID‐19, including tachyarrhythmias, respiratory failure, AKI, and the requirement of invasive mechanical ventilation [6–8]. COVID‐19 can cause cardiac injury through multiple mechanisms, including cardiac injury because of acute and severe inflammatory response secondary to a cytokine storm, viral invasion of cardiomyocytes, or ischemic injury due to hypoxia from acute lung injury [9]. Hence, because of the cardiac sequelae of COVID‐19, and based on the experience of CHD patients with previous viral illnesses like influenza and RSV, pediatric patients with cardiac conditions remain a vulnerable group, highlighting the need to evaluate effective therapeutic strategies in this group [9].

Several pharmacological treatments have been used against COVID‐19, falling into the following classes: antiviral agents like lopinavir/ritonavir, inflammation inhibitors like tocilizumab and anakinra, low‐molecular‐weight heparins, and plasma. Furthermore, extensive efforts have been directed toward developing effective vaccines against COVID‐19, which is challenged by the rapidly mutating SARS‐CoV‐2 [10]. Remdesivir, the focus of this study, is a nucleoside analog that competitively inhibits viral RNA‐dependent RNA polymerase (RdRp), with documented activity against a broad spectrum of viruses [11, 12]. It is of particular interest because of its widespread use against COVID‐19 and the conflicting results concerning its efficacy [13]. It is typically given once daily for a period of 5–10 days as an intravenous infusion to reduce time to recovery and reduce the risk of progression to severe COVID‐19 [12].

SARS‐CoV‐2 affects all age groups and presents with several symptoms. In the pediatric population, COVID‐19’s severe clinical presentation is less common than that in adults but still possible. The vast majority of COVID‐19 cases among the pediatric population are asymptomatic, mild severity, or moderate severity, with the most severe cases being among infants [14]. Fever and cough are the most reported symptoms of COVID‐19 among children. Moreover, most pediatric cases of COVID‐19 had a history of close contact with a diagnosed family member [15]. A small proportion of patients may develop a severe post‐infectious inflammatory illness known as multisystem inflammatory syndrome in children (MIS‐C), which is an immune dysfunction following an acute infection. In patients with MIS‐C, fever, abdominal pain, diarrhea, and vomiting are the most common manifestations [16].

While the pediatric population is generally underexplored when it comes to COVID‐19, there is also a great shortage of studies on the appropriate treatment of COVID‐19 among children. Although remdesivir was among the first antiviral agents to be authorized for use in treating COVID‐19, its use in pediatric cardiac patients, who may be especially vulnerable to drug‐related complications [13, 17], is heavily under‐investigated. Because the existing literature is highly fragmented and often nonspecific to the pediatric population, there is a significant limitation on evidence‐based decision‐making in the high‐risk population of pediatric cardiac patients. Due to a lack of comprehensive analysis of the safety and efficacy of COVID‐19 drugs in cardiac pediatric patients in the existing literature, clinical decisions are often made in the absence of robust patient‐specific evidence, with the rationale typically extrapolated from the general population despite the recognized heterogeneity of CHD and documented interindividual variability in response to drugs in general. Children with CHD frequently exhibit altered drug pharmacokinetics and pharmacodynamics, predisposing them to suboptimal therapeutic responses, adverse drug reactions, and clinically significant drug–drug interactions, with these risks further compounded by the high burden of hospitalizations attributable to medication‐related events [18]. Hence, this literature review first provides an overview of remdesivir’s development and mechanism of action. Then, utilizing data from randomized controlled trials, observational studies, case series, and case reports, it aims at exploring remdesivir’s clinical application in COVID‐19 while highlighting its potential risks, particularly in the pediatric cardiac population.

## 2. Methodology

The literature search was conducted on PubMed and Google Scholar until May 4, 2025. Initially, the following keywords and Medical Subject Headings (MeSH) terms were combined as follows: (“remdesivir” [Supplementary Concept] OR “remdesivir” OR “GS‐5734”) AND (“COVID‐19”[Mesh] OR “SARS‐CoV‐2”[Mesh] OR “COVID‐19” OR “COVID 19” OR “SARS‐CoV‐2” OR “2019‐nCoV”). The initial search yielded 4499 records, reflecting the extensive volume of COVID‐19‐related literature and widespread investigation of remdesivir across diverse populations, study designs, and outcomes. Scope‐limiting filters were initially applied to view case reports, clinical and randomized controlled trials, meta‐analyses, and observational studies, yielding 836 results. Subsequently, a more detailed search was conducted, looking into remdesivir’s cardiac side effects using the AND operator, yielding 382 results. Altogether, 119 duplicates were identified. Given the narrative nature of the review and its focus on pediatric and cardiac outcomes, targeted searches within this set were conducted in stages, yielding a subset of records that underwent title, abstract, and full‐text review. Background references were identified separately and were not part of the remdesivir‐focused screening process. The inclusion criteria included articles that studied populations of patients who had confirmed COVID‐19 and where remdesivir was used as a part of the treatment protocol. This study excludes any ongoing clinical trials and only reviews papers written in or translated into English. This narrative review was not conducted according to a predefined systematic review protocol, and no formal risk‐of‐bias assessment was performed.

## 3. Results

### 3.1. Drug Development

Remdesivir (GS‐5734) first emerged to treat RNA‐based viruses and particularly Ebola, followed by members of the *Coronaviridae* family. Although previously known as potent therapeutic agents for RNA viruses, nucleoside analogs are poorly cell‐permeable. Hence, modified nucleosides, like the monophosphate form of GS‐441524, known as GS‐5734 or remdesivir, were utilized and found to be highly effective against RNA viruses, including the common cold human coronaviruses, MERS, and SARS. Administered intravenously, remdesivir has a loading dose of 200 mg in adults, followed by a maintenance dose of 100 mg daily for up to 10 days, adjusted for body weight in the pediatric population [19].

### 3.2. Mechanism of Action

**FIGURE 1:**
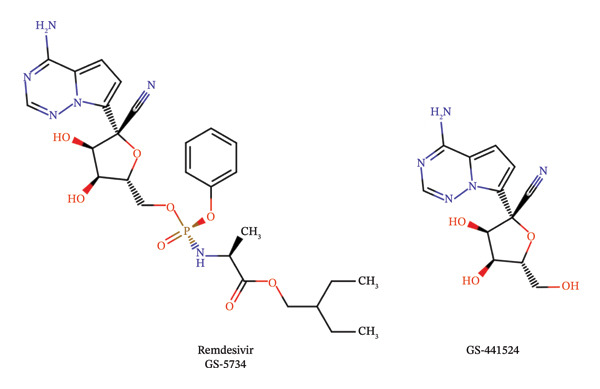
Chemical structure of remdesivir and its metabolite. Created in Marvin JS by ChemAxon.

**FIGURE 2:**
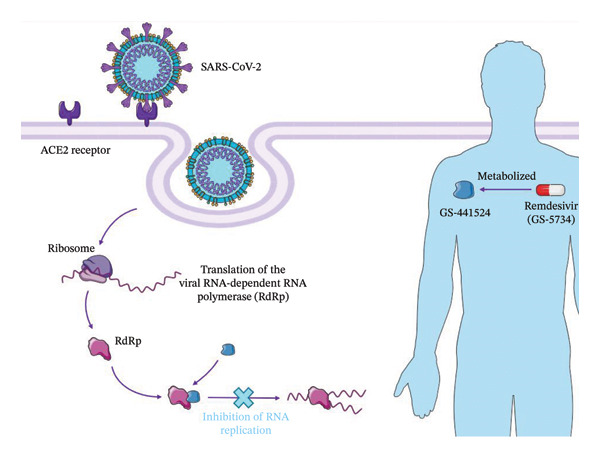
Remdesivir’s mechanism of action. Assets provided by Servier Medical Art (https://smart.servier.com/), licensed under CC BY 4.0 (https://creativecommons.org/licenses/by/4.0).

Antivirals targeting RdRp include nucleoside analogs, which fall into three categories: mutagenic nucleotides, obligate chain terminators, or delayed chain terminators. Remdesivir is a delayed chain terminator, which has a 3′OH, meaning it can form a phosphodiester bond when the chain is elongating, but blocks transcription due to the 1’‐CN subunit, which sterically hinders the RdRp and prevents further chain elongation. Remdesivir (GS‐5734) is the prodrug form of GS‐441524, which can more easily permeate into the cell. Refer to Figure 1 for the structures of each. Intracellularly, remdesivir is modified by hydrolysis of the amino acid ester to yield an alanine metabolite (GS‐704277). This alanine metabolite is ultimately modified into the nucleoside triphosphate (NTP) that is misused by the RdRp, leading to inhibition of viral replication. In short, remdesivir’s antiviral activity occurs through sterically interacting with the viral RdRp due to its 1′‐CN group, which is effective against multiple RNA viruses [19]. Figure 2 summarizes remdesivir’s mechanism of action.

### 3.3. Randomized Controlled Trials

While remdesivir is heavily explored in its use against COVID‐19, results from randomized controlled trials, summarized in Table 1, conflict in terms of the clinical significance of its use. Additionally, most studies were conducted during the peak time of the pandemic, meaning the progress of the research process might have been repeatedly interrupted to protect the research staff from being exposed to COVID‐19 [20]. Most of the clinical trials used a uniform IV loading dose of 200 mg on the first day of the treatment, followed by 100 mg per day.

**TABLE 1:** Randomized controlled trials on the use of remdesivir.

Study	Date and location	Design	Population	Intervention and primary endpoint	Conclusion	Limitations
Remdesivir for the Treatment of COVID‐19—Final Report [21]	United States, Denmark, the United Kingdom, Greece, Germany, Korea, Mexico, Spain, Japan, and Singapore, 2020	Randomized, double‐blind, placebo‐controlled trial	1062 adults were admitted to the hospital for COVID‐19 with confirmed lower respiratory tract infection	Intervention: Remdesivir (200 mg on the first day with 100 mg for up to 9 days) or placebo (for up to 10 days) Primary endpoint: Time to recover.	Remdesivir contributes to lowering the time to recovery, lowering mortality, and decreasing the possibility of COVID‐19 adverse effects	1. Restricted travel during the time of this trial 2. Hospitals restricted the access of nonessential research team members 3. Lack of adequate personal protective equipment and trial‐related supplies in some centers
Early Remdesivir to Prevent Progression to Severe COVID‐19 in Outpatients [23]	United States, Spain, Denmark, and the United Kingdom, 2021	Randomized, double‐blind, placebo‐controlled trial	567 nonhospitalized patients with COVID‐19 who had at least one risk factor for disease progression (age ≥ 60 years, obesity, or certain coexisting medical conditions)	Intervention: remdesivir for 3 days (200 mg on the first day followed by 100 mg daily for 2 additional days) or placebo Primary endpoint: Any adverse event associated with COVID‐19	A 3‐day course of remdesivir had an acceptable safety profile and is associated with an 87% lower risk of hospitalization or death compared to placebo.	1. High‐risk coexisting conditions and certain races are underrepresented 2. Conducted primarily in the United States 3. Included only 8 adolescents 4. Excluded patients who took the COVID‐19 vaccines, making it difficult to generalize these results 5. Conducted before the emergence of the Delta variant 6. The trial achieved less than half of the planned enrollment rates because it was stopped early for administrative reasons
Remdesivir for the Treatment of Patients in Hospital with COVID‐19 in Canada: A Randomized Controlled Trial [22]	Canada, 2022	Open‐label, randomized controlled trial	1282 adult patients admitted to the hospital with laboratory‐confirmed SARS‐CoV‐2 infection	Intervention: remdesivir for 10 days (200 mg on the first day followed by 100 mg for 9 days) with standard care, or standard care alone Primary endpoint: in‐hospital mortality	In comparison with standard care alone, the combination of remdesivir and standard care together improved secondary outcomes (like the need for mechanical ventilation)	1. Limited power to prove statistical significance on the primary mortality outcome 2. Information of the specific infecting variant for each patient is unavailable, so the effect of remdesivir on different strains is impossible to evaluate 3. Could not contact a portion of patients post‐treatment for follow‐up and outcome assessment
Clinical Antiviral Efficacy of Remdesivir in Coronavirus Disease 2019: An Open‐Label, Randomized Controlled Adaptive Platform Trial (PLATCOV) [24]	Thailand and Brazil, 2023	Multicenter, open‐label, controlled, adaptive, pharmacometrics platform trial	Low‐risk adult patients with early symptoms of COVID‐19	Intervention: 200 mg remdesivir followed by 100 mg/day for 5 days or no treatment Primary endpoint: Rate of SARS‐CoV‐2 clearance	Parenteral remdesivir enhances viral clearance in early symptomatic COVID‐19	1. The relationship between the rate of viral clearance and therapeutic efficacy is not well established. 2. Used a 5‐day course of remdesivir, although a 3‐day course is approved in some countries 3. Open‐label study, which may have led to biases in reporting adverse symptoms
Effect of Remdesivir vs. Standard Care on Clinical Status at 11 Days in Patients With Moderate COVID‐19: A Randomized Clinical Trial [25]	United States, Europe, and Asia, 2020	Randomized, open‐label trial	596 patients with confirmed SARS‐CoV‐2 infection and moderate pneumonia	Intervention: 10‐day course of remdesivir, 5‐day course of remdesivir, or standard care (200 mg the first day followed by 100 mg/day) Primary endpoint: clinical status on Day 11 on a 7‐point ordinal scale^1^	Among patients with moderate COVID‐19, those on a 10‐day course of remdesivir did not have a significant difference in clinical outcome with standard care at 11 days after treatment initiation. Patients on a 5‐day course had a statistically significant difference in clinical status compared with standard care, but with uncertain clinical importance.	1. Change in the primary endpoint they planned to study, from the hospital discharge rates, which varied greatly across regions, to a standardized ordinal scale^1^ 2. Open‐label design might have led to biases 3. Effect of remdesivir on viral load was not assessed 4. Limitations of the ordinal scale used^1^
Remdesivir in Adults With Severe COVID‐19: A Randomized, Double‐Blind, Placebo‐Controlled, Multicenter Trial [28]	China, 2020	Randomized, double‐blind, placebo‐controlled, multicenter trial	237 adults admitted with COVID‐19. Patients had an interval between symptom onset and enrollment of 12 days or less, oxygen saturation of 94% or less, ratio of arterial oxygen partial pressure to fractional inspired oxygen of 300 mg or less, and radiologically confirmed pneumonia	Intervention: Remdesivir for 10 days (200 mg on the first day followed by 100 mg for 9 days) or placebo infusions for 10 days Primary endpoint: Time to clinical improvement within 28 days.	No statistically significant clinical benefit for remdesivir	1. Insufficient power to establish differences in clinical outcome 2. Initiation of remdesivir treatment late in COVID‐19 3. Lack of virological data (recovery from the infectious virus) 4. Frequent use of corticosteroids in the study sample
Clinical Outcomes of Using Remdesivir in Patients with Moderate to Severe COVID‐19: A Prospective Randomized Study [27]	India, 2021	Randomized controlled trial	82 patients who were more than 40 years old with moderate to severe COVID‐19 confirmed by a PCR assay within the last 4 days. Patients were not on mechanical ventilation	Intervention: remdesivir for 5 days with standard care, or only standard care Primary endpoint: Improvement in clinical outcomes (clinical status on Day 14, time to clinical improvement, recovery, and death from any cause)	No statistically significant difference in the final treatment outcome of patients in the two groups. Remdesivir for 5 days did not produce significant improvement in clinical outcomes in moderate to severe COVID‐19 cases	1. All patients studied were moderate to severe cases. However, the disease is progressive, so the definitions of “moderate” and “severe” are variable 2. No grading of the adverse events 3. No placebo was given in the no‐remdesivir group, and no blinding 4. Shortage of supporting staff during peak pandemic 5. Single‐center with small sample size
Remdesivir Plus Standard of Care vs. Standard of Care Alone for the Treatment of Patients Admitted to Hospital with COVID‐19 (DisCoVeRy): A Phase 3, Randomized, Controlled, Open‐Label Trial [29]	France, Belgium, Austria, Portugal, Luxembourg, 2021	Phase 3, open‐label, adaptive, multicenter, randomized, controlled trial	824 adult patients who were admitted to the hospital with laboratory‐confirmed COVID‐19 infection and illness of any duration. Patients also had clinical evidence of hypoxemic pneumonia or required oxygen supplementation	Intervention: receive standard care alone or in combination with remdesivir, lopinavir–ritonavir, lopinavir–ritonavir and interferon beta‐1a, or hydroxychloroquine Primary endpoint: clinical status at Day 15 measured by the seven‐point ordinal scale^1^	Among patients who were symptomatic for more than 7 days and required oxygen support, there was no clinical benefit from the use of remdesivir	1. Open‐label. Not placebo controlled 2. Multiple different types of treatments were given at the beginning of the trial 3. No viral load assessment for 18% of the patients 4. Plasma concentrations of remdesivir and its metabolite GS‐441524 were assessed in only 10% of participants, and concentrations of intracellular active metabolites were not measured
Remdesivir and Three Other Drugs for Hospitalized Patients with COVID‐19: Final Results of the WHO Solidarity Randomized Trial and Updated Meta‐Analyses [30]	35 countries, 2021	Randomized controlled trial, open label	8275 hospitalized patients with definite COVID‐19 subdivided by disease severity (indicated by the use of supplementary oxygen and ventilation)	Intervention: remdesivir daily IV 200 mg on Day 0 and 100 mg on Days 1–9, or no study drug Primary endpoint: in‐hospital mortality	There was no significant benefit to patients already on ventilation. In other hospitalized patients, there was only a modest reduction in the risk of death or need for ventilation.	1. Reasons for oxygen requirements not documented 2. In some countries, ventilation was more resource‐limited 3. Patient recruitment occurred prior to Delta and Omicron variants and extensive vaccination 4. Controls received no placebos 5. Study size
Remdesivir for COVID‐19 in Hospitalized Children: A Phase 2/3 Study [41]	Italy, Spain, the United States, and the United Kingdom, 2024	Phase 2/3, open‐label trial	53 children between the ages of 28 days and 17 years. Hospitalized for positive PCR assay of SARS‐CoV‐2.	Intervention: remdesivir treatment for 10 days or less, with once‐daily IV remdesivir doses defined based on pharmacokinetic modeling using body weight measurements. Primary endpoint: The fraction of patients with treatment‐emergent adverse events, laboratory abnormalities, and plasma pharmacokinetics of remdesivir and its metabolites	Remdesivir is associated with improvement in clinical status, similar drug exposure in pediatric patients compared to adults, and no new safety concerns	1. Single‐arm study, with small numbers of participants in each cohort. 2. Comparisons between cohorts and generalizations to larger populations are limited 3. Preexisting medical histories of patients limit generalizability

^1^The WHO ordinal scale was [1]: not hospitalized, no limitations on activities [2]; not hospitalized, limitation on activities [3]; hospitalized, not requiring supplemental oxygen [4]; hospitalized, requiring supplemental oxygen [5]; hospitalized, on noninvasive ventilation or high‐flow oxygen devices [6]; hospitalized, on invasive mechanical ventilation or extracorporeal membrane oxygenation [7]; death.

Some randomized controlled trials ascertain remdesivir’s efficacy against COVID‐19. Beigel et al. conducted a study including 1062 hospitalized adults to assess the effect of remdesivir on time to recovery from COVID‐19. Among the participants, the recovery time was reduced in the group given remdesivir compared to the placebo, along with a reduced mortality and lower risk of developing adverse events of COVID‐19 [21]. Ali et al. similarly found remdesivir to reduce outcomes such as in‐hospital mortality, clinical severity, oxygen or mechanical ventilation use, duration of hospital stay, and adverse event rates [22]. Gottlieb et al. conducted a randomized controlled trial on 562 symptomatic nonhospitalized patients and concluded that remdesivir reduced the risk of hospitalization or death compared to placebo, with an acceptable safety profile [23]. Similarly, Ali et al.’s findings support this conclusion. Finally, while there is no established relationship between viral clearance and therapeutic efficacy, Jittamala et al.’s study conducted on low‐risk adult patients with COVID‐19 concluded that parenteral remdesivir enhances viral clearance of COVID‐19 [24].

Multiple studies suggest that the use of remdesivir has no significant effect in treating COVID‐19. Spinner et al. studied 596 hospitalized patients with moderate COVID‐19 pneumonia to examine the clinical status of patients after a 10‐day course of remdesivir, a 5‐day course, or after standard care treatment. The study concluded that a 10‐day remdesivir treatment is not statistically different from the 5‐day treatment regimen, but those on the 5‐day course had a statistically significant difference in clinical status compared to those on standard care, although the clinical importance is doubtful because inequalities in patient care at multiple sites were not controlled for [25]. The non‐inferiority of a 5‐day course was corroborated by Goldman et al., albeit their population was not representative of critically ill patients [26]. However, Mahajan et al., who studied a similar population, found that even 5 days of treatment did not lead to significant improvement [27], supported by Wang et al.’s findings, which did not prove a statistically significant benefit for the use of remdesivir over a 10‐day course [28]. Ader et al. conducted their multicenter randomized controlled trial across Europe on 824 adult patients admitted to the hospital for COVID‐19 with evidence of pneumonia or who required oxygen supplementation. They found that in patients who had been symptomatic for over 7 days and required supplemental oxygen, remdesivir did not provide any observable clinical benefit [29]. Finally, the WHO Solidarity trial, a large‐scale international trial involving 14,304 hospitalized patients, of which 8275 were studied using remdesivir versus control, concluded that there was no mortality reduction in patients already on ventilation but some reduction, albeit with wide confidence intervals, in patients receiving oxygen but not being ventilated [30].

The majority of the randomized controlled trials evaluating the efficacy of remdesivir for COVID‐19 have been conducted exclusively on the adult population. Almost none of the trials addressed the pediatric population or paid any particular attention to populations with underlying cardiac diseases. Hence, evidence‐based decision‐making concerning the use of remdesivir in pediatric cardiac patients remains highly unsupported.

### 3.4. Compassionate Use Studies

Compassionate program studies, summarized in Table 2, were also conducted after Gilead Sciences started accepting requests from clinicians for compassionate use of remdesivir for adults on January 25, 2020 [31] and for children on March 21, 2020 [32]. Grein et al.’s study on 61 patients on mechanical ventilation or extracorporeal mechanical oxygenation found that 68% of the patients illustrated clinical improvement [31]. Antinori et al. enrolled 35 eligible patients with multiple coexisting conditions, ranging from obesity to cancer. The status of 88.2% of the patients improved by Day 28 from the date of starting remdesivir treatment. However, there was a 44.4% case fatality rate among participants who were started on the remdesivir treatment in the intensive care unit (ICU). The researchers concluded that the remdesivir treatment can be of benefit in treating patients with COVID‐19 pneumonia hospitalized outside of the ICU, where adverse effects were minimal [33]. Finally, Goldman et al. delivered remdesivir through a compassionate‐use program to 77 hospitalized COVID‐19 patients under 18 years old with numerous comorbidities like asthma, prematurity, and hematologic and neurologic conditions for a 10‐day course. The majority of the patients in the study recovered regardless of the baseline need for mechanical ventilation [32]. Even with the focus on observational studies, the high‐risk pediatric cardiac population remains underrepresented. Hence, because evidence behind the use of antivirals in the pediatric cardiac population is hugely reliant on adult data, there is an underlying need to examine the available pediatric data separately.

**TABLE 2:** Compassionate care use of remdesivir.

Study	Date and location	Design	Population	Intervention and primary endpoint	Conclusion	Limitations
Compassionate Use of Remdesivir for Patients with Severe COVID‐19 [31]	United States, Europe, Canada, Japan, 2020	Compassionate‐use program	61 patients with confirmed SARS‐CoV‐2 infection with an oxygen saturation of 94% or less or who are receiving oxygen support	Remdesivir for 10 days (200 mg on the first day followed by 100 mg/day for 9 days) Primary endpoint: No preset primary outcome. They quantified the incidence of key clinical events like changes in oxygen‐support requirements, hospital discharge, adverse events, and death	Improvement in oxygen‐support status was observed in 68% of patients	1. Small size of cohort 2. Short duration of follow‐up 3. Potential missing data 4. Lack of information on 8 of the patients initially treated
Compassionate Remdesivir Treatment of Severe COVID‐19 Pneumonia in Intensive Care Unit (ICU) and Non‐ICU Patients: Clinical Outcome and Differences in Post‐Treatment Hospitalization Status [33]	Italy, 2020	Prospective (compassionate), open‐label study of remdesivir	Adult patients with SARS‐CoV‐2 pneumonia undergoing mechanical ventilation with an oxygen saturation of 94% or less or a National Early Warning score of 2 of 4 or more	Intervention: 10‐day course of remdesivir (200 mg on Day 1 followed by 100 mg/day for 9 days) Primary endpoint: change in clinical status based on a 7‐category ordinal scale^1^	Remdesivir is associated with better clinical outcomes and fewer adverse events in patients with SARS‐CoV‐2 pneumonia hospitalized out of the ICU	1. Most patients included in the study were previously treated with LPV/r + HQC 2. Could not conclude the virological efficacy of remdesivir in clearing viral RNA in patients′ respiratory samples
Compassionate Use of Remdesivir in Children With Severe COVID‐19 [32]	United States, Spain, United Kingdom, Italy, France, Germany, 2021	Compassionate‐use program	77 hospitalized patients under 18 years old with confirmed SARS‐CoV‐2 infection	Intervention: For children of 40 kg or more, a loading dose of 200 mg was used on Day 1, followed by 100 mg/day. For children less than 40 kg, a loading dose of 5 mg was used on Day 1, followed by 2.5 mg/day. Recommended treatment for 10 days. Primary endpoint: Clinical outcomes (safety, respiratory support status, hospital discharge, and recovery)	Most of the children studied recovered with a low rate of adverse effects	Short duration of follow‐up (28 days)

^1^The WHO ordinal scale was [1]: not hospitalized, no limitations on activities [2]; not hospitalized, limitation on activities [3]; hospitalized, not requiring supplemental oxygen [4]; hospitalized, requiring supplemental oxygen [5]; hospitalized, on noninvasive ventilation or high‐flow oxygen devices [6]; hospitalized, on invasive mechanical ventilation or extracorporeal membrane oxygenation [7]; death.

### 3.5. Remdesivir in the Pediatric Population

Clinical trials on pediatric patients are extremely limited [34]. Hence, many of the decisions on pediatric patient treatments come from observational studies, retrospective analyses, or extrapolation from adult data, summarized in Table 3. Overall, studies have shown that remdesivir is safe for use in pediatric patients, whether previously healthy or with comorbidities [34–38]. Mendez‐Echevarria et al. conducted an observational compassionate use study on 9 pediatric patients. After studying the progression of illness by measuring the number of days from remdesivir treatment to viral clearance, liver enzymes, and renal impairment, the authors illustrated that most of the cohort arrived at successful clinical outcomes without adverse effects [39]. This was corroborated by Samuel et al.’s retrospective study involving 48 hospitalized pediatric patients. Studying remdesivir’s efficacy through assessing for bradycardia, clinical improvement, hypertension, AKI, and drug‐induced liver injury, the authors found that remdesivir use correlated with clinical status improvement [37]. Khalil et al. similarly found lower rates of hospitalization, a lower proportion of adverse events, and a lower requirement of respiratory support among those treated with remdesivir [34]. Cocuz et al.’s retrospective study on 14 pediatric patients with COVID‐19 of different severities found that remdesivir led to clinical improvement and nonprogression of disease [38]. Kautsch et al.’s retrospective study on 328 pediatric patients showed that in patients with relatively milder disease, the drug significantly reduced the risk of disease progression. However, in those with severe COVID‐19, remdesivir did not contribute to a reduced mortality rate, contrasting with Cocuz et al.’s study [40].

**TABLE 3:** Observational studies on the use of remdesivir in the pediatric population.

Study	Date and location	Design	Study endpoint	Conclusion	Limitations
Compassionate Use of Remdesivir in Children with COVID‐19 [39]	Spain, 2020	Observational study on 9 pediatric patients who received compassionate treatment with remdesivir	Clinical outcome of patients as indicated by mean days from remdesivir treatment to viral clearance, liver enzymes, and renal impairment	Most of the cohort arrived at successful clinical outcomes, without adverse events.	1. Small size of cohort 2. Many patients received the drug when the disease had progressed
Safety of Remdesivir in 20 Children with COVID‐19—Case Series—[35]	Japan, 2022	Case series of 20 hospitalized pediatric patients	Clinical outcomes of pediatric patients as measured by [1] patients’ background [2] time from onset of symptoms, or a positive COVID test, to the start of remdesivir treatment [3]; duration of remdesivir treatment [4]; other treatments [5]; clinical course: symptoms [6] adverse events	Remdesivir is safe for use in pediatric patients against COVID‐19	1. Each physician had the freedom to choose the use of antibiotics 2. Each physician had the freedom to choose the duration of treatment with remdesivir 3. The study is not variant‐specific
Evaluation of the Safety Profile and Therapeutic Efficacy of Remdesivir in Children with SARS‐CoV‐2 Infection—A Single‐Center, Retrospective, Cohort Study [40]	Poland, 2023	Retrospective cohort study on 328 children hospitalized for COVID‐19	Safety and efficacy of remdesivir in pediatric patients hospitalized with COVID‐19	Remdesivir is safe to use for children with COVID‐19, but the efficacy is still questionable. For children with mild or asymptomatic disease or with risk factors for severe disease, remdesivir is effective. Nevertheless, its efficacy against severe disease is doubtful.	Small size of study group
Remdesivir Use in Pediatric Patients for SARS‐CoV‐2 Treatment: Single Academic Center Study [37]	United States, 2022	Retrospective study on 48 pediatric patients	Clinical improvement, bradycardia, hypertension, acute kidney injury, and drug‐induced liver injury	Remdesivir may be related to clinical stability or improvement in pediatric patients	1. Unable to establish causality or statistical significance 2. Confounding factors like simultaneous treatment with dexamethasone 3. Small patient population
Efficacy and Safety of Remdesivir in Hospitalized Pediatric COVID‐19: A Retrospective Case–Controlled Study [34]	Qatar, 2023	Retrospective case–controlled study on 60 pediatric patients	Clinical symptoms associated with remdesivir treatment (URTIs, CNS effects, GI symptoms)	Remdesivir is well‐tolerated in pediatric patients and may be a safe therapeutic option for COVID‐19	1. Small sample size 2. Single‐institution study
Treatment with Remdesivir of Children with SARS‐CoV‐2 Infection: Experience from a Clinical Hospital in Romania [38]	Romania, 2024	Retrospective study of 14 patients	Clinical status of pediatric patients as measured by improvement and discharge from hospital, oxygen administration, severity of COVID‐19, and the need for antibiotic or cortisone treatment	Appropriate use of remdesivir is efficient and safe in the pediatric population	1. Single‐center study 2. Small sample size 3. Sometimes the medical records were incomplete
Remdesivir Use in Pediatric Patients with Acute SARS‐CoV‐2 Infection Is Safe and Well Tolerated [36]	United States, 2025	Single‐center retrospective cohort study on 318 pediatric patients	Safety of remdesivir use in COVID‐19 pediatric patients as measured by discharge alive, discharge on oxygen, pediatric intensive care unit admission, invasive ventilatory support, length of in‐hospital stay, unplanned discontinuation of treatment, and reason for discontinuation	Remdesivir is well‐tolerated in patients under 19 years old	Absence of control arm

Among extremely rare randomized controlled trials done on the pediatric population is the phase 2/3 CARAVAN study conducted by Ahmed et al. on the use of remdesivir on 53 children, where they concluded that remdesivir was associated with improvement in clinical status with no new major safety concerns. Additionally, the pharmacokinetic profiles of remdesivir in this pediatric population were comparable to those observed in adults. However, this study was a single‐arm study with no control groups, meaning conclusiveness is doubtful [41]. Despite the few studies reporting results on the pediatric population, the results pertaining to the pediatric cardiac population are almost absent from the current literature.

### 3.6. Side Effects

Multiple side effects to remdesivir were reported in adults. Constipation, hypoalbuminemia, hypokalemia, anemia, thrombocytopenia, increased total bilirubin, elevated liver function tests, nausea, cough, and headache were among the major adverse effects [23, 25, 28, 33, 35]. As a result of serious to moderate side effects, including respiratory failure or ARDS, in Wang et al.’s study, more patients in the remdesivir group discontinued the study compared to the placebo group. However, none of the deaths were judged to be related to the intervention [28]. This is unlike Ader et al.’s findings on 824 participants, where three of the deaths were attributed to remdesivir. Furthermore, the occurrence of side effects, mainly acute respiratory failure, ARDS, and AKI, was not significantly different between the placebo and the remdesivir group [29]. In a compassionate use program study conducted by Grein et al., 60% of patients reported adverse events, while 23% of patients had serious adverse events, most commonly septic shock, AKI, and multiple‐organ dysfunction. 8% of patients discontinued the treatment for multiple reasons, some of which were attributable to comorbidities at baseline [31]. Moreover, another similar study showed that the most common adverse event that necessitated the discontinuation of the study was AKI, most of whom eventually died [33].

Spinner et al. found that adverse events were experienced by patients in the 5‐day remdesivir group and the 10‐day remdesivir group, although more prominently in the 10‐day group. Adverse events were rarer in the remdesivir group as opposed to the standard care group, and deaths were not attributed to the remdesivir treatment [25]. In Goldman et al.’s study, the percentage of patients experiencing side effects in the 5‐day group and the 10‐day group was similar, unlike in Spinner et al.’s study. However, 10% of the patients in the 10‐day group, as opposed to 4% of the patients in the 5‐day group, discontinued the treatment, with more serious adverse effects being more common among the 10‐day group [32]. Such findings conflict with two other studies, where a higher percentage of adverse events were reported in the control group [23, 24]. In further contradistinction, three other studies find that adverse events were not significantly different between the remdesivir group and the standard of care group [22, 27, 41].

Adverse effects in children are similar to those in adults, including elevated liver enzymes and PT, vomiting, neutropenia, hypokalemia, hypoalbuminemia, anemia, and thrombocytopenia [34, 35]. Increases in liver enzymes were mild and did not require discontinuation of treatment [36, 38, 40]. No adverse events were attributed to the use of remdesivir in a retrospective study on 48 pediatric patients. About 20% of the patients experienced bradycardia, and approximately half experienced hypertension, but none required discontinuation of the study. None experienced renal or hepatic toxicity. Interestingly, one patient developed bilateral arm tremors while receiving remdesivir with no other known contributing factors to this tremor, but it resolved on its own. One of the patients died 7 months after hospitalization when they were receiving their remdesivir treatment, but the patient had a complicated history, including seizure disorders and chronic aspirations. Hence, there is no clear proof that this event is due to remdesivir, although it remains possible due to remdesivir’s cardiac side effects [37].

### 3.7. Use in the Pediatric Cardiac Population

While COVID‐19 infection in the pediatric population is generally assumed to be milder than that in adult patients, it comes with the risk of cardiac side effects, especially in patients with CHD [42]. Particularly, myocarditis, arrhythmias, cardiogenic shock, and MIS‐C are among the most important forms of cardiac involvement among healthy pediatric patients with COVID‐19 [43].

Remdesivir’s cardiotoxic effects are generally underexplored in COVID‐19 patients. After remdesivir was licensed for use, Jung et al. conducted a retrospective pharmacovigilance cohort study and found the main cardiac adverse events reported to be cardiac arrest, bradycardia, shock, hypotension, atrial fibrillation, and acute myocardial infarction after controlling for multiple potential confounders [44], corroborated by another pharmacovigilance study [45]. In an early study on safety and heart rate changes associated with remdesivir, significant heart rate reduction was found, but with no symptomatic bradycardia, arrhythmias, or QTc prolongation necessitating discontinuation of remdesivir [46]. Other studies report different findings. One historical cohort of 322 patients found a significantly prolonged QT following 5 days of administration of remdesivir, although the difference in QTc was not found to be significant [47]. Another case–control study similarly found QT prolongation in 20.4% of patients taking remdesivir, along with bradycardia in 8.8%, both of which were significantly higher than in the control group [48].

Multiple case reports note these cardiotoxic effects. A case report on a 37‐year‐old man with COVID‐19 who was started on dexamethasone and remdesivir developed asymptomatic sinus bradycardia at 40–44 beats/min a few hours after receiving the second dose of remdesivir. Upon discontinuation of remdesivir, the heart rate improved. Hence, the author suggests a baseline ECG should be performed for patients before receiving remdesivir, along with continuous monitoring during treatment [49]. Similarly, a case report on a 59‐year‐old man was published in 2021. The patient developed bradycardia after 4 days of remdesivir treatment with a heart rate reaching 50 beats/min, which then returned to normal after stopping remdesivir [50]. Mehannek et al. also report two adult cases who developed bradycardia following remdesivir use. One patient initially developed symptomatic bradycardia that persisted asymptomatically following cessation of remdesivir. The other patient initially developed QT prolongation alongside bradycardia that resolved on discharge but reappeared later and persisted on follow‐up visits. Of note, this patient has a history of bradycardia that has resolved [51]. The case of a 13‐year‐old boy with episodic asthma was reported, whereby he was diagnosed with bilateral pneumonia with hypoxemia due to SARS‐CoV‐2 infection. He was started on remdesivir, and after the third dose, he developed an asymptomatic and non‐hemodynamically significant sinus bradycardia confirmed via an ECG. Upon cessation of remdesivir, the heart rate returned to its normal range [52]. Finally, a case report on two adolescents with complex CHD developed rapid‐onset hypotension, conduction abnormalities, and refractory cardiogenic shock within 3 hours of remdesivir administration, aligning with the drug’s pharmacokinetics, ultimately resulting in death [53].

The pathophysiological mechanism was explored using an in vitro approach by Kwok et al., who exposed human induced pluripotent stem cell‐derived cardiomyocytes (hiPSC‐CMs) to remdesivir under two conditions, normoxic and hypoxic, to mimic mild and severe COVID‐19. They showed that remdesivir induced mitochondrial and nonmitochondrial fragmentation that persisted beyond the cessation of treatment [54]. Katja et al. similarly noted damaged cell proliferation and energy metabolism due to mitochondrial toxicity, as shown by reduced oxygen consumption and increased lactate secretion. This causes a functional impairment in situations where tissue repair or renewal is needed, in addition to an effect on the heart’s contractile function due to impaired energy metabolism, explaining the bradycardia reported in patients after remdesivir use [55]. Another notion to consider is the fact that remdesivir is an adenosine analog, which might cause blocking effects at the AV node similar to those induced by adenosine [52].

Hence, taken together, these reported cardiotoxic effects of remdesivir necessitate careful consideration prior to prescription, particularly in patients with pre‐existing cardiac abnormalities. Furthermore, noting that pediatric patients with pre‐existing cardiovascular conditions are prone to more severe COVID‐19 presentations [56], the safety and efficacy of remdesivir in this high‐risk population require targeted evaluation to avoid over‐prescribing this medication and exacerbating cardiac symptoms.

## 4. Discussion

Through synthesizing the current evidence surrounding the use of remdesivir in the treatment of COVID‐19, this narrative review reveals that, while remdesivir was among the earliest antivirals to be authorized for use in COVID‐19, the overall body of evidence reveals several inconsistencies. While compassionate‐use programs of remdesivir significantly contributed to physicians’ understanding of remdesivir’s role in COVID‐19, these findings needed to be confirmed by randomized controlled trials. Such trials conducted on adult patients have demonstrated mixed results regarding efficacy, where subsequent large‐scale trials, including the WHO Solidarity trial, failed to show a significant benefit in mortality [30]. Similarly, the 2023 Cochrane review concluded that remdesivir likely has little or no effect on mortality for adults hospitalized with moderate to severe COVID‐19, albeit it may slightly improve clinical outcomes, such as increasing the chance of discharge and reducing the risk of clinical worsening (e.g., the need for invasive ventilation or death). However, this evidence is of low certainty. While the evidence is of limited certainty, remdesivir possibly reduces the risk of hospitalization or worsening disease for nonhospitalized or worsening disease [57].

Although there are other treatment options for COVID‐19, such as monoclonal antibodies like bamlanivimab and casirivimab/imdevimab, remdesivir remains one of the few drugs proven effective in multicenter, placebo‐controlled, randomized controlled trials. Even with that in mind, further randomized trials including strictly pediatric patients are also needed to retrieve high‐quality events and ensure effective medical care, especially for the high‐risk population of pediatric cardiac patients [58]. Additionally, with the rapid emergence of the SARS‐CoV‐2 Omicron variant, the global effort to develop Omicron‐specific vaccines illustrates how viral evolution can substantially alter the effectiveness of interventions [59]. Similar concerns apply to antiviral agents like remdesivir. However, despite the genetic variation of SARS‐CoV‐2 and their rapid spread, studies on nearly 6 million variant isolates found that Nsp12, the target of remdesivir, is relatively conserved, preserving remdesivir’s potency against most of the SARS‐CoV‐2 viruses and thus indicating sustained use of remdesivir against the SARS‐CoV‐2 tested variants [60]. If resistance develops, alternatives include increased dosages of remdesivir, compounds with a similar mechanism of action that display increased affinity, or the utilization of drugs with a different therapeutic target [12].

Early guideline statements recommended supportive care alone for most COVID‐19 cases in pediatrics since it usually follows a mild course. For children with severe illness (i.e., requiring supplementary oxygen but not noninvasive or invasive mechanical ventilation or extracorporeal membrane oxygenation or ECMO), remdesivir was suggested. For critically ill patients, meaning those requiring noninvasive or invasive mechanical ventilation, a 5‐day course of remdesivir should also be considered [61]. More recent data support these statements. Due to the limited number of randomized controlled trials conducted in the pediatric population, data stems largely from observational studies. These studies have yielded less conflicting information than that among adults, where most studies suggest remdesivir’s efficacy and safety. However, such evidence must be interpreted with caution due to the small sample sizes and lack of randomized comparisons. Nevertheless, the CARAVAN study, which suggested remdesivir, when initiated early in the disease course, may contribute to clinical improvement in children with moderate to severe COVID‐19, offers promising results [41]. These require further elucidation by other randomized controlled trials.

While most studies report remdesivir to be generally well‐tolerated, due to the potential to be metabolized to a lesser extent by hepatic enzymes, it should not be used in individuals with severe liver disease. Furthermore, one of remdesivir’s metabolites—GS‐441524—is renally cleared; hence, remdesivir is not recommended in patients with impaired kidney function, especially those with a glomerular filtration rate below 30 mL/min [58]. Remdesivir in children was found to have a similar pharmacokinetic profile to adults [41] and hence warrants the same precautions. Additionally, due to the potential of cardiac side effects, a baseline ECG may be warranted before initiating remdesivir therapy to establish cardiac conduction norms and assess for increased risk, accompanied by ongoing monitoring throughout the course of treatment [49, 51]. This may be especially true in patients with electrolyte disturbances, renal dysfunction, structural heart disease, heart failure, or advanced age, or potentially taking QT‐prolonging medications [47, 48]. Further studies are required to elucidate the mechanisms behind these cardiotoxic effects.

There is a marked heterogeneity observed in the results of multiple randomized controlled trials evaluating the use of remdesivir in COVID‐19. This could be due to the differences in the population studied, especially when it comes to differences in disease severity at baseline, whereby some studies targeted hospitalized patients [22] generally, while others targeted patients with specific exacerbations like hypoxemic pneumonia [29], for example, and others targeted nonhospitalized patients [23]. This could have caused differences in the efficacy of remdesivir at different points during the course of the illness. In addition, this heterogeneity could be attributed to the differences in the primary endpoints, which included time to recovery [21], the development of adverse events [23], and the rate of mortality of patients [22]. Finally, the experience the medical staff gained during the pandemic meant that their capacity to handle COVID‐19 cases likely improved, potentially yielding better outcomes as time passed.

There is an unmet need for data in specific pediatric subpopulations—particularly, cardiac patients, who are at higher risk of adverse effects of COVID‐19 and are additionally burdened by altered cardiovascular physiology, limited cardiac reserve, and frequent exposure to polypharmacy [42]. Remdesivir’s cardiac side effects, ranging from bradycardia to cardiogenic shock, are reported in various forms of literature. Altered hemodynamics, chronic ventricular volume or pressure overload, and reduced myocardial reserve may influence drug distribution and clearance, increasing the susceptibility of CHD patients to adverse cardiovascular effects [62]. Hence, these potential side effects necessitate the continuous monitoring of cardiac performance among pediatric patients, especially those with an underlying disease that may predispose them to a worsened COVID‐19 prognosis and, therefore, be more likely to use remdesivir and be exposed to its potential cardiotoxicity [52]. Furthermore, as remdesivir appears to confer its greatest clinical benefit when administered early in the course of infection, once the therapeutic window has passed, it would be prudent to approach its use in critically ill children with CHD with caution due to the persisting myocardial susceptibility. As reducing the dose would diminish antiviral efficacy, treatment deferral may be appropriate in select high‐risk patients [53]. Finally, current pediatric dosing strategies are primarily weight‐based and do not account for underlying cardiac physiology or myocardial vulnerability. While the prolongation of infusion duration has been proposed to reduce peak‐related toxicity, available evidence suggests that remdesivir‐associated myocardial effects may persist beyond 24 h, limiting the protective impact of such adjustments [53]. Pharmacogenomic profiling offers a promising future method to inform clinical decisions in CHD patients by providing genetic information on how patients metabolize specific drugs, thereby reducing adverse drug effects, making dosing more precise, and avoiding treatment failure [18].

These observations underscore a critical gap in the management of COVID‐19 in pediatric patients, in that there is an absence of physiologically informed dosing strategies and cardiac‐specific safety data for remdesivir in children with CHD. Given that drug‐related adverse effects account for a substantial proportion of hospitalizations among pediatric cardiac patients, caution is warranted when extrapolating adult or noncardiac pediatric data to this population [18]. In select high‐risk scenarios, particularly in critically ill children with complex CHD, active systemic inflammation, or marginal cardiac reserve, the potential risks of remdesivir may outweigh its uncertain benefits, and withholding therapy may be a reasonable consideration.

## 5. Conclusion

The COVID‐19 pandemic necessitated rapid evaluation of multiple antiviral therapies across various populations, especially vulnerable populations like pediatric patients with cardiac conditions. While remdesivir is currently approved for use against COVID‐19, there is a clear imbalance in the evidence available for its use in the literature, where some studies reported an acceptable safety and efficacy of remdesivir while others reported a statistically insignificant improvement in the clinical course of COVID‐19 upon using remdesivir. Aside from the marked heterogeneity in the literature, remdesivir’s use in children, particularly those with cardiac comorbidities, remains less studied. While randomized controlled trials remain the most valuable source of validation for the use of certain drugs, these are extremely limited in the context of the pediatric population, resulting in a reliance on retrospective studies and data extrapolated from adult populations, which in themselves are heterogeneous and inconclusive. Studies addressing the use of antivirals in pediatric cardiac patients with COVID‐19 are particularly necessary because of the increased severity of COVID‐19 outcomes, measured by the need for supplemental oxygen or in‐hospital death, in patients with preexisting cardiovascular conditions, especially among children younger than 12 years [56]. The randomized controlled trials and case reports published on the pediatric population are extremely limited and thus inconclusive in terms of the safety of remdesivir, particularly in pediatric cardiac patients. Findings from limited studies on the pediatric population suggest that remdesivir, when dosed correctly, may achieve protective effects for the pediatric population. The emerging discussion of the cardiotoxic effects of remdesivir maximizes the need to explore the safety considerations of using remdesivir in the pediatric cardiac population. In conclusion, while early data may support the cautious use of remdesivir in pediatric patients with underlying cardiac conditions, more specific, large‐scale studies are needed to establish conclusive results about its safety and efficacy. Future research should prioritize prospective pediatric studies focused on the pediatric population with congenital or acquired cardiac conditions to assess the cardiac safety and variant‐specific outcomes. Until such evidence becomes available, clinical decisions must be based on potential benefits versus individualized cardiac risk.

## Author Contributions

Dima Bsat and Dalia Safi contributed to the conceptualization of the review and wrote the initial draft. Dima Bsat constructed the tables, and Dalia Safi constructed the figures. Mariam Arabi conceptualized and supervised the project.

## Funding

No funding was received for this research.

## Conflicts of Interest

The authors declare no conflicts of interest.

## Data Availability

Data sharing is not applicable to this article as no new data were created or analyzed in this study.
